# Abscisic acid alleviates chilling injury in cold-stored peach fruit by regulating ethylene and hydrogen peroxide metabolism

**DOI:** 10.3389/fpls.2022.987573

**Published:** 2022-09-06

**Authors:** Jixing Tang, Yaoyao Zhao, Shuning Qi, Qi Dai, Qiong Lin, Yuquan Duan

**Affiliations:** ^1^Key Laboratory of Agro-products Quality and Safety Control in Storage and Transport Process, Ministry of Agriculture and Rural Affairs, Institute of Food Science and Technology, Chinese Academy of Agricultural Sciences, Beijing, China; ^2^Department of Food Science, Shenyang Agricultural University, Shenyang, China

**Keywords:** *Prunus* *persica*, chilling injury, ascorbate-glutathione cycle, hydrogen peroxide, ethylene metabolism

## Abstract

Peach (*Prunus persica* (L.) Batsch) is susceptible to chilling injury under improper low-temperature storage (2°C–5°C). Previous research has shown that abscisic acid (ABA) alleviates chilling injury in fruits and vegetables, but the potential mechanism is still unclear. To explore its effectiveness and potential mechanism in alleviating chilling injury during cold storage, exogenous ABA was applied to peach fruit by immersion in 100 μmol L^−1^ solutions for 10 min. In our experiment, ABA alleviated chilling injury by reducing hydrogen peroxide (H_2_O_2_) content and ethylene production. In addition, ABA inhibited the expression of the ethylene synthesis-related genes *PpACO1* and *PpEIN2*. At the same time, ABA activated the antioxidant enzymatic pathway and the ascorbate-glutathione (AsA-GSH) cycle, the transcript abundance encoding genes related to antioxidant enzyme activities also changed correspondingly. The results suggested that ABA alleviated chilling injury by scavenging excessive H_2_O_2_ by promoting antioxidant enzymes and the AsA-GSH pathway.

## Introduction

Peach [*Prunus persica* (L.) Batsch] is a typical climacteric fruit that ripens and rots quickly at ambient temperature after harvest. Low-temperature storage is a common method of storing and transporting peaches. However, peaches are sensitive to chilling injury during cold storage, especially from 2.2°C to 7.6°C (Lurie and Crisosto, 2005). The symptoms of chilling injury include browning or woolly texture of the flesh, reduced ethylene release, increased decay susceptibility, and abnormal ripening (Lurie and Crisosto, 2005). The chilling injury severely affects commercial value and prompts quality deterioration (Yao et al., 2021; Islam et al., 2022; Zhu et al., 2022). Therefore, investigating proper methods to alleviate chilling injury, is important for maintaining the commercial value of peach fruit.

As a critical phytohormone, ethylene is a key factor that regulates the ripeness and senescence of peach fruit. In addition to being highly correlated with fruit softening, ethylene is also associated with postharvest metabolic disorders and rot (Netlak et al., 2021). Previous research has recently confirmed that immature fruits stored at low temperatures affected ethylene synthesis by altering the expression of ethylene metabolism-related genes (Megías et al., 2016). Candan et al. (2006) have confirmed the widespread involvement of ethylene in regulating cold tolerance in postharvest fruit. 1-methylcyclopropane (1-MCP), a typical inhibitor of ethylene, can effectively alleviate chilling injury symptoms of climacteric fruits such as persimmon (Kou et al., 2020), plums (Menniti et al., 2004), and peach (Liu et al., 2018) by inhibiting the signal transfer of ethylene. In addition, previous research indicated that peach fruit treated with Jasmonic acid can reduce chilling injury symptoms by regulating ethylene metabolism (Zhao et al., 2021a). Ethylene has been proved associated with abnormal ripening and softening during refrigeration storage. Therefore, how to regulate ethylene production may be a very critical factor in alleviating chilling injury.

Postharvest fruit ripening and senescence are considered to be highly linked to oxidative damage. When plants are exposed to adversity stresses, such as low temperature, leading to the imbalance of reactive oxygen species (ROS) metabolism (Babalar et al., 2018; Yang et al., 2021). Excessive accumulation of ROS accumulations eventually leads to oxidative damage (Zhou et al., 2014), such as DNA damage, lipid peroxidation, and finally results in cell death. The prevailing view is that the excessive accumulation of ROS causes the decline of quality in postharvest fruits and vegetables (Meitha et al., 2020). The natural antioxidant defense system present in plants helps them to scavenge ROS, thus reducing oxidative damage. Several enzymatic antioxidant pathways contain superoxide dismutase (SOD), peroxidase (POD), and catalase (CAT). SOD can convert O^2−^·into H_2_O_2_ (Lounifi et al., 2012). Thereafter, the effect of POD and CAT decomposed H_2_O_2_ to H_2_O. AsA-GSH cycle was another important section of reactive oxygen scavenging system consisting of some typical enzymes like ascorbate peroxidase (APX), glutathione peroxidase (GPX), and glutathione reductase (GR). Ascorbate peroxidase also contributed to the degradation of H_2_O_2_. Previous study showed that the regulation of the antioxidant system could enhance drought stress tolerance (Hasan et al., 2021), heat tolerance (Tao et al., 2020), toxicity defense (Altaf et al., 2021), and cold tolerance in many plants, such as phalaenopsis seedlings (*Phalaenopsis aphrodite* H. G. Reichenbach; Chen and Ko, 2021), mung bean (*Vigna radiata* L.; Nahar et al., 2015) and cucumber (*Cucumis sativus* L.; Zhang et al., 2019).

Abscisic acid (ABA) as a natural plant hormone, regulates several physiological functions including stomatal closure, leaf abscission, senescence, fruit ripening and so on (Parwez et al., 2022). At present, there are numerous studies related to molecular mechanisms of ABA biosynthesis. Abscisic acid biosynthesis involves many crucial enzymes such as Zeaxanthin epoxidase (ZEP), 9′ cis epoxycarotenoid dioxygenase (NCED) and abscisic aldehyde oxidase (AAO; Zhou et al., 2021). Abscisic acid is widely involved in responding to various adversities and stresses, and it is one of the important regulators of chilling injury mitigation (Ciura and Kruk, 2018). There is the evidence that exogenous application of ABA can effectively alleviate chilling injury of vegetables and fruits. For example, zucchini squash fruit storage under high relative humidity (HRH) conditions alleviates chilling injury by promoting the accumulation of endogenous ABA (Zuo et al., 2021). Abscisic acid can effectively reduce internal browning (IB) in pineapple (Zhang et al., 2015) and maintain the color and quality of postharvest grapes (Cantín et al., 2007). An increasing amount of literature is devoted to explain that improved antioxidant capacity is associated with fruit quality, such as lemon (*Citrus limon* (L.) Burm. f.; Zhang and Zhou, 2019), goji (*Lycium barbarum* L.; Zhang et al., 2021), sweet cherry (*Prunus avium* L.; Zhao et al., 2019), and Rosa sterilis fruit (*R. sterilis*; Dong et al., 2022). In this result, we found that ABA treatment exactly improved the activity of antioxidant enzymes consistent with slight chilling injury symptoms during cold storage. However, resistance to cold stress is not exclusively controlled by a single ABA signal, but often acts in concert with other regulatory factors and the mechanism needs to be deeply explored.

Based on the above background, we assessed the effect of ABA on essential enzymes related to ROS metabolism and analyzed transcript abundance accordingly. Insight to explore the deep mechanism of ABA in alleviating chilling injury and offer an effective strategy to preserve postharvest peach fruit.

## Materials and methods

### Plant materials and sampling

The peaches (*Prunus persica L. Batsch*., “Jinqiuhongmi”) were picked from an orchard in Weifang, Shandong Province, China, after they reached an eighth mature stage, which means that the peel of green color has faded and turned white, and the flesh is fuller and less fuzz. On the same day, the fruit was delivered to the Chinese Academy of Agricultural Science (CAAS) lab. Each group contained 150 fruits, consisting of three replicates of 50 fruit each. One of the following solutions was used to soak the peaches for 10 min: (1) distilled water (control); (2) 100 μmol L^−1^ abscisic acid (Solarbio Life Sciences, Beijing, China). Then all fruit were stored at 4°C for 5 weeks under 90%–95% relative humidity. Three biological replicates containing three fruit each were used for the analysis of quality and biochemistry at 0, 7, 14, 21, 28, and 35 days following harvest. After the physiological measurement, fruit slices from each of the three replicates were mixed and stored at −80°C for the following experiment.

### Evaluation of internal browning index

The evaluation criteria of chilling injury are IB. The fruit was cut along the axial diameter and assessed chilling injury visa visual IB of each fruit. The calculation of the IB index is referenced to Wang et al. (2018).

The severity of the chilling injury was estimated by the scale of the IB region of peach fruit and scored from 0 to 4 as follows:

0, nearly no browning; 1, browning scale was 1%–25%; 2, browning scale was 26%–50%; 3, browning scale was 51%–75%; and 4, browning scale was 76%–100%. The chilling injury index was calculated using the formula: chilling injury index = [(chilling injury score) × (number of fruits with this chilling injury score)]/(4 × total number of fruits in each treatment).

### Measurement of ethylene production

Ethylene production was calculated as follows: Select six fruit per treatment from the refrigerator house and divide them into three groups at each sampling time point. Each treatment group was placed in a 1.5 L sealed container for 2 h at room temperature, the ethylene production was measured following the methods described by Zhao et al. (2021a).

### Measurement of respiration rate

For each treatment, the respiration rate was recorded following the methods described by Song et al. (2021).

Six peach fruit at each sampling point were composed 2 fruits in each group in three replicates, and they were sealed in 1.5-L boxes for 2 h, using a portable CO_2_ infrared analyzer (F950, Felix Instruments) to measure the total amount of CO_2_ and calculate the respiration rate based on the total amount of CO_2_.

### Measurement of H_2_O_2_ content

Hydrogen peroxide (H_2_O_2_) content was measured using the H_2_O_2_ content detection kit (Solarbio, BC3590-50T/48S, Beijing, China), taking 0.1 g of powdered sample and adding the extraction solution. The homogenized sample was then centrifuged at 8,000 *g* for 10 min at 4°C, strictly following the instructions.

### Measurement of enzymes activities related to antioxidant enzyme pathway and AsA-GSH pathway

SOD, POD, APX, and GR activities in peach peel tissues were measured using a POD assay kit (Solarbio, BC0090-50T/48S, Beijing, China), a SOD assay kit (Solarbio, BC0175-100T/48S, Beijing, China), an APX assay kit (Solarbio, BC0220-50T/48S, Beijing, China), and a GR assay kit (Solarbio, BC1160-50T/48S, Beijing, China), respectively, based on the guidelines of the manufacturer, taking 0.1 g of powdered sample and adding the extraction solution. The homogenized sample was then centrifuged at 8,000 *g* for 10 min at 4°C, strictly following the instructions.

### Total RNA extraction and RNA-seq

Total RNA was isolated according to acetyltrimethylammonium bromide (CTAB) method with little modifications (Zhao et al., 2021a). At the same time, considering that the chilling symptoms of peach fruit occurred 21 days after ABA treatment, fruits were selected after 21 days storage as the time points for RNA-seq. The samples were labeled as CK_21A, CK_21B, CK_21C, ABA_21A, ABA_21B, and ABA_21C. Libraries for RNA sequencing (RNA-Seq) were prepared according to Zhao et al. (2022). RNA-seq was performed on an Illumina HiSeq 2500 sequence platform at ouyi company (Shanghai, China). The adaptor sequences and low-quality sequence reads were first removed from the data sets completely (Bolger et al., 2014), then the paired and clean reads were mapped to the peach genome.^1^ Transcript abundance level was expressed by Fragments Per Kilobase Million (FPKM). Differential expression analysis was performed using DESeq2 software. Genes with fold change >2 and false discovery rate (FDR) < 0.05 were regarded as differentially expressed genes (DEGs). The specific calculation is referred to in our previous study (Zhao et al., 2022).

### Reverse transcription-quantitative PCR (RT-qPCR) analysis

cDNA synthesis was referred to the previous method with slight modifications for real-time quantitative PCR (Zhao et al., 2021a). cDNA was generated using A SYBR Green Q-PCR Kit (Takara, RR420A, Japan) following the manufacturer’s guidelines and qRT-PCR was completed using the Applied Biosystems 7,500 Fast Real-Time PCR System (Thermo Fisher Scientific, United States). According to Livak and Schmittgen (2001), relative gene expression was calculated using the Comparative 2^−ΔΔCT^ method. Primer sequences can be found in Supplementary Tables S1, S2.

### Statistical analysis

SPSS version 17.0 was used for all statistical analyses, and the figures were prepared using Origin 8.6. Differences between control and treated fruit were assessed with significance at *p* < 0.05.

## Results

### Effect of ABA on chilling injury index of peach fruit

**Figure 1:**
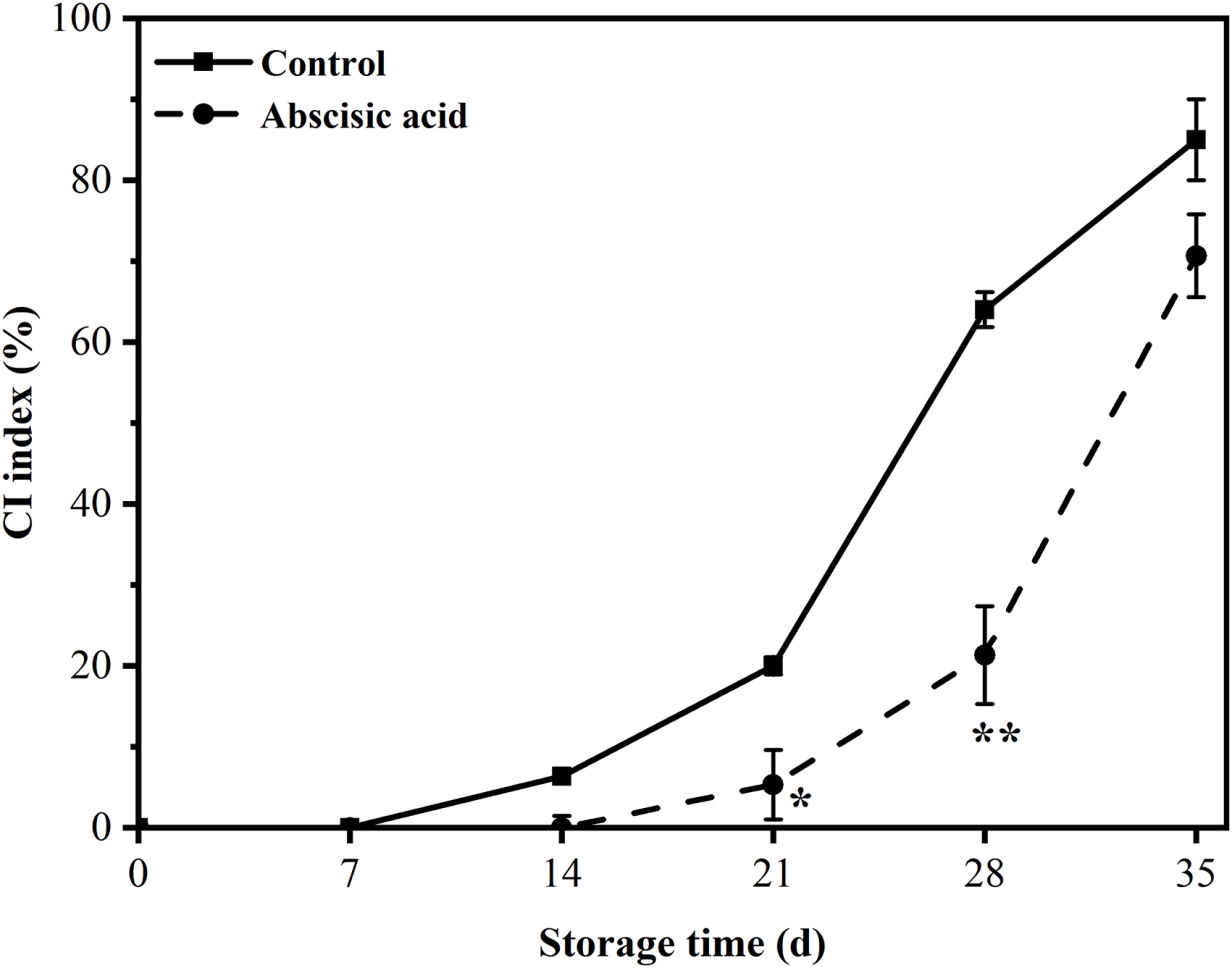
Effect of abscisic acid (ABA) treatment on chilling injury index of peach fruit during cold storage at 4°C. Vertical bars represent the standard deviation of the means of triplicate samples (*n* = 3). The symbol (*) indicates significant differences among different treatments at *p*   <  0.05. The symbol (**) indicates significant differences among different treatments at *p*  <  0.01.

To assess whether the alleviation of chilling injury was affected by ABA, we examined the IB region. Examples of IB are shown in Figure 1, the control fruit demonstrated slight chilling injury symptoms after 14 days of storage. On the contrary, IB nearly did not appear in ABA-treated fruit at day 14, but a little appeared at day 21. The chilling injury index in the control fruit was ~4 times and 3 times that of ABA-treated fruit, respectively, at day 21 and day 28, but during the end of storage, the chilling injury index of the two groups was very close. According to this result, we concluded that ABA was effective in reducing chilling injury symptoms during cold storage (*p* < 0.05). However, the effect of ABA treatment beyond 4 weeks of storage was not significant, especially at day 35, peach fruit showed severe IB and lost commodity value absolutely.

### Effect of ABA on respiration and ethylene production rate of peach fruit

**Figure 2:**
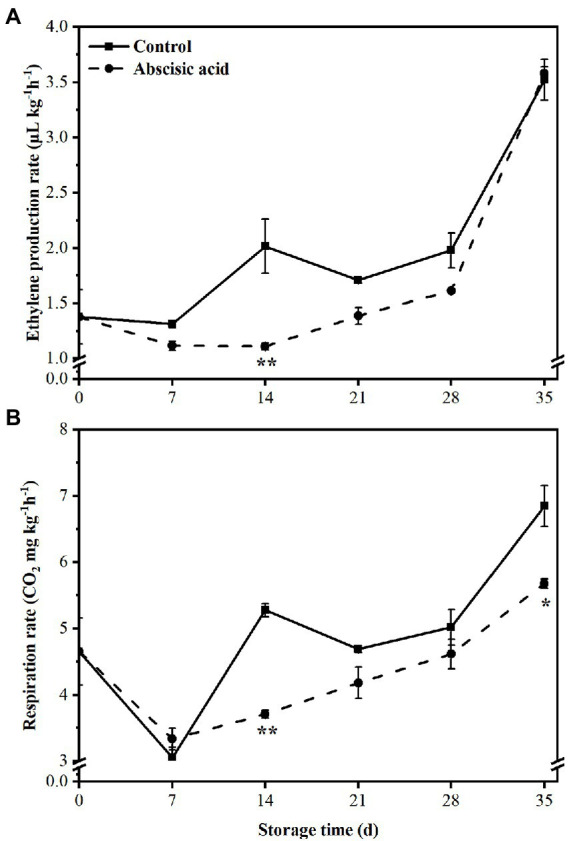
Effect of abscisic acid (ABA) treatment on ethylene production **(A)** and respiration rate **(B)** of peach fruit during cold storage at 4°C. Vertical bars represent the standard deviation of the means of triplicate samples (*n* = 3). The symbol (*) indicates significant differences among different treatments at *p*   <  0.05. The symbol (**) indicates significant differences among different treatments at *p*  <  0.01.

Levels of ethylene production remained relatively stable between the control and ABA-treated fruit for the first 7 days. After that, ethylene production by both the control and ABA-treated fruit increased rapidly; application of ABA was effective in reducing ethylene production at day 14 (Figure 2A; *p* < 0.05), and there was no difference in later stages of storage. At the end of the storage, ABA treatment nearly keep the same value of ethylene production compared with the CK fruit.

Respiration rate showed a similar trend both in treated fruit and control fruit during the entire storage period. At first, respiration rate declined sharply in the first 7 days of storage and then increased gradually regardless of treatment. However, treatment with ABA significantly (*p <* 0.05) inhabited the respiration rate at day 14 and day 35 (Figure 2B). The significant reduction in ABA-treated fruit reached 30% and 17%, respectively, compared with that in control fruit (*p* < 0.05).

### Effect of ABA on H_2_O_2_ content of peach fruit

**Figure 3:**
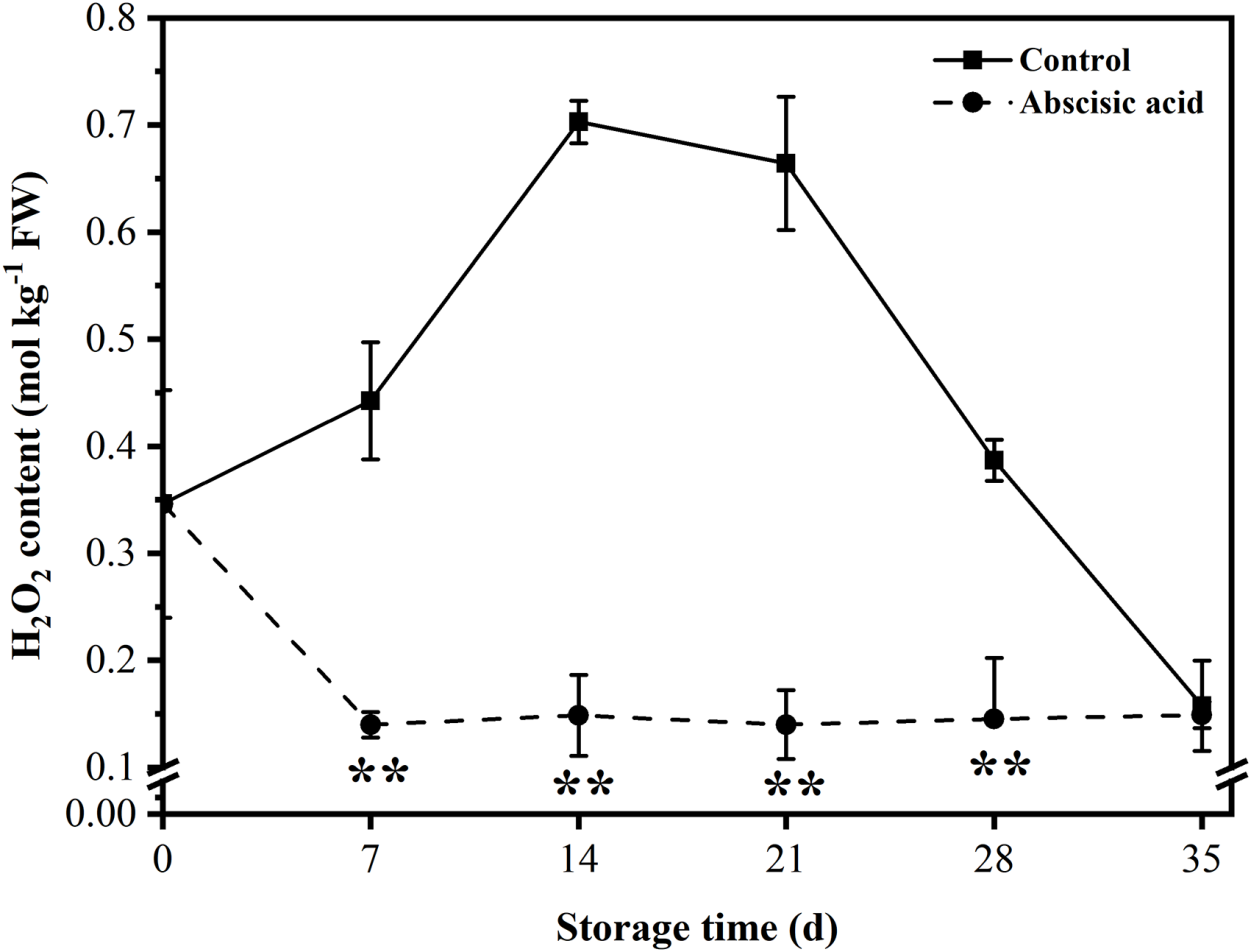
Effect of abscisic acid (ABA) treatment on H_2_O_2_ content of peach fruit during cold storage at 4°C. Vertical bars represent the standard deviation of the means of triplicate samples (*n* = 3). The symbol (**) indicates significant differences among different treatments at *p*  <  0.01.

As shown in Figure 3, ABA treatment reduced the H_2_O_2_ content in peach fruit. H_2_O_2_ content rose sharply at first and reached a peak at day 14, but the content of H_2_O_2_ in ABA-treated fruit decreased at day 7. In the following storage period, H_2_O_2_ content in control exhibited a decreasing trend during this period, as for ABA-treated fruit, the H_2_O_2_ content was at a relatively stable value during the next 4 weeks. It is worth noting that ABA significantly reduced the H_2_O_2_ content, especially during the first 4 weeks of refrigeration period (*p* < 0.05). It is suggested that a significant function of ABA is to reduce the level of H_2_O_2_ content, thus lessening peroxide damage to cells and maintaining the balance of ROS metabolisms in plants.

### Effect of ABA on the expression of genes related to ethylene synthesis and its signaling pathway

**Figure 4:**
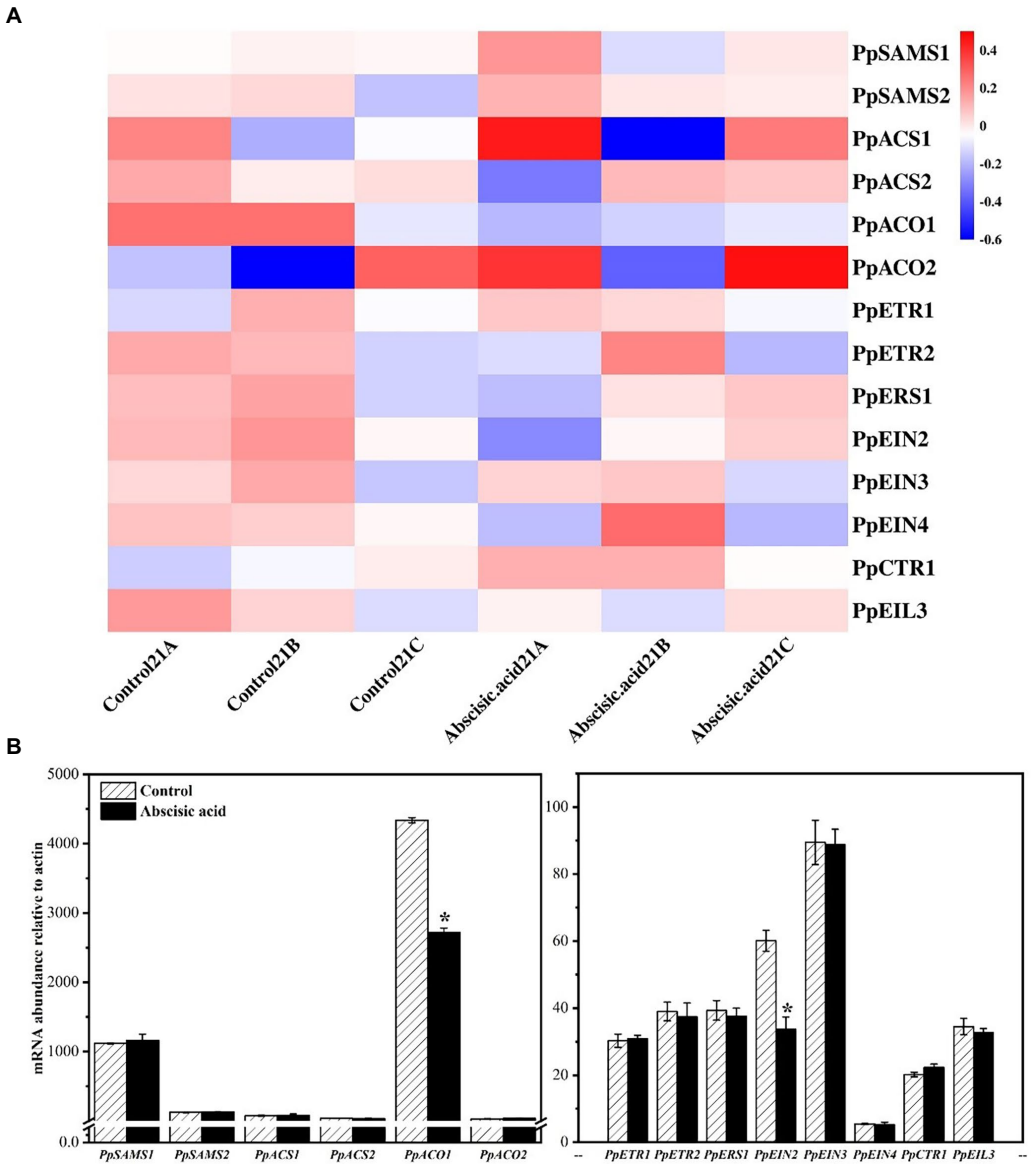
Effect of abscisic acid (ABA) treatment on the mRNA abundance of genes related to ethylene biosynthesis and the ethylene signaling pathway in fruit stored at 4°C. **(A)** Heat map showing transcript abundances of differentially expressed genes as determined by RNA-seq. **(B)** Validation of mRNA abundances as determined using RT-qPCR. Data are means of three biological replicates ±SE. The symbol (*) indicates significant differences among different treatments at *p*  <  0.05.

We have analyzed the transcript abundance of all genes by RNA-seq (Supplementary Figure 1). After 21 days of low-temperature storage, ABA significantly inhibited the expression of *PpACO1* (*p* < 0.05), which is one of the important members of ethylene synthesis. The results of RT-qPCR (Figure 4B) and RNA-seq were basically the same. In addition, we also examined genes involved in the ethylene signaling pathway (Figure 4B). The data show that gene expression of ethylene receptors *PpEIN2* was significantly inhibited by ABA.

### Effect of ABA on the gene expression of the antioxidant enzyme pathway and the AsA-GSH pathway

**Figure 5:**
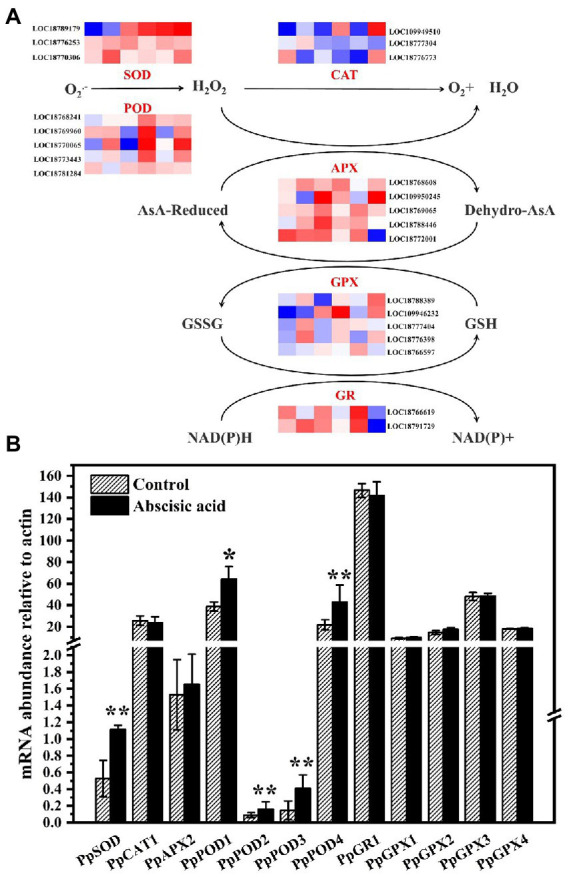
**(A)** Effects of abscisic acid (ABA) treatment on the expression profiles of genes associated with reactive oxygen species metabolic pathways in peach fruit stored at 4°C. The rows in each heat map represent the indicated genes, and the six columns indicate the following storage times (left-to-right): CK_21dA, CK_21dB, CK_21dC, ABA_21dA, ABA_21dB, and ABA_21dC. The colors represent the FPKM values between different samples in the heat map. Each value represents the mean for three replicates. SOD, superoxide dismutase; POD, peroxidase; CAT, catalase; APX, ascorbate peroxidase; GPX, glutathione peroxidase; GR, glutathione reductase, monodehydroascorbate reductase. **(B)** Validation of mRNA abundances of sugar metabolism genes as determined using RT-qPCR. Data are means of three biological replicates ± SE. The symbol (*) indicates significant differences among different treatments at *p*   <  0.05. The symbol (**) indicates significant differences among different treatments at *p*  <  0.01.

Plants have a sophisticated metabolic network for ROS. In a bid better to investigate the underlying mechanism of exogenous ABA regulating ROS metabolism in peach fruit, we examined several genes related to antioxidant enzymes including *PpSODs*, *PpPODs*, *PpCATs*, *PpAPXs*, *PpGPXs*, *and PpGRs.* SOD can catalyze the dismutation of O_2_^−^ to H_2_O_2_. Figure 5B showed that *PpSOD* expression level of the control was relatively lower during the cold storage. In addition, the relative expression of *PpSOD* of ABA treatment was significantly up-regulated (*p* < 0.05), and it was ~2.13 times that of control. CAT and POD can catalyze H_2_O_2_ and oxidize phenols. *PpPOD* was annotated by five genes (LOC18768241, LOC18769960, LOC18770065, LOC18773443, and LOC18781284). The expression levels for all five genes exhibited an upward trend during the cold storage. As shown in Figure 5B, the expression level of *PpPOD* was markedly increased by ABA treatment at day 21 (*p* < 0.05). *PpCATs* (LOC109949510, LOC18777304, and LOC18776773) expression abundance in ABA-treated fruit both increased and decreased at day 21 (Figure 5B). *PpAPXs* and *PpGPXs* are, respectively, annotated by (LOC18768608, LOC109950245, LOC18769065, LOC18788446, and LOC18772001) and (LOC18788389, LOC109946232, LOC18777404, LOC18776398, and LOC18766597). Moreover, the five *PpAPXs* had different expression patterns. The relative expression of *PpAPX2* of ABA treatment was up-regulated, but there was no difference between control and ABA treatment. In addition, the rest of the genes were down-regulated by ABA treatment (Figure 5B). As for *PpGPXs*, compared with CK, all five genes were induced by ABA at day 21 (Figure 5B).

### Effect of ABA on the enzyme activities of the antioxidant enzyme pathway and the AsA-GSH pathway

**Figure 6:**
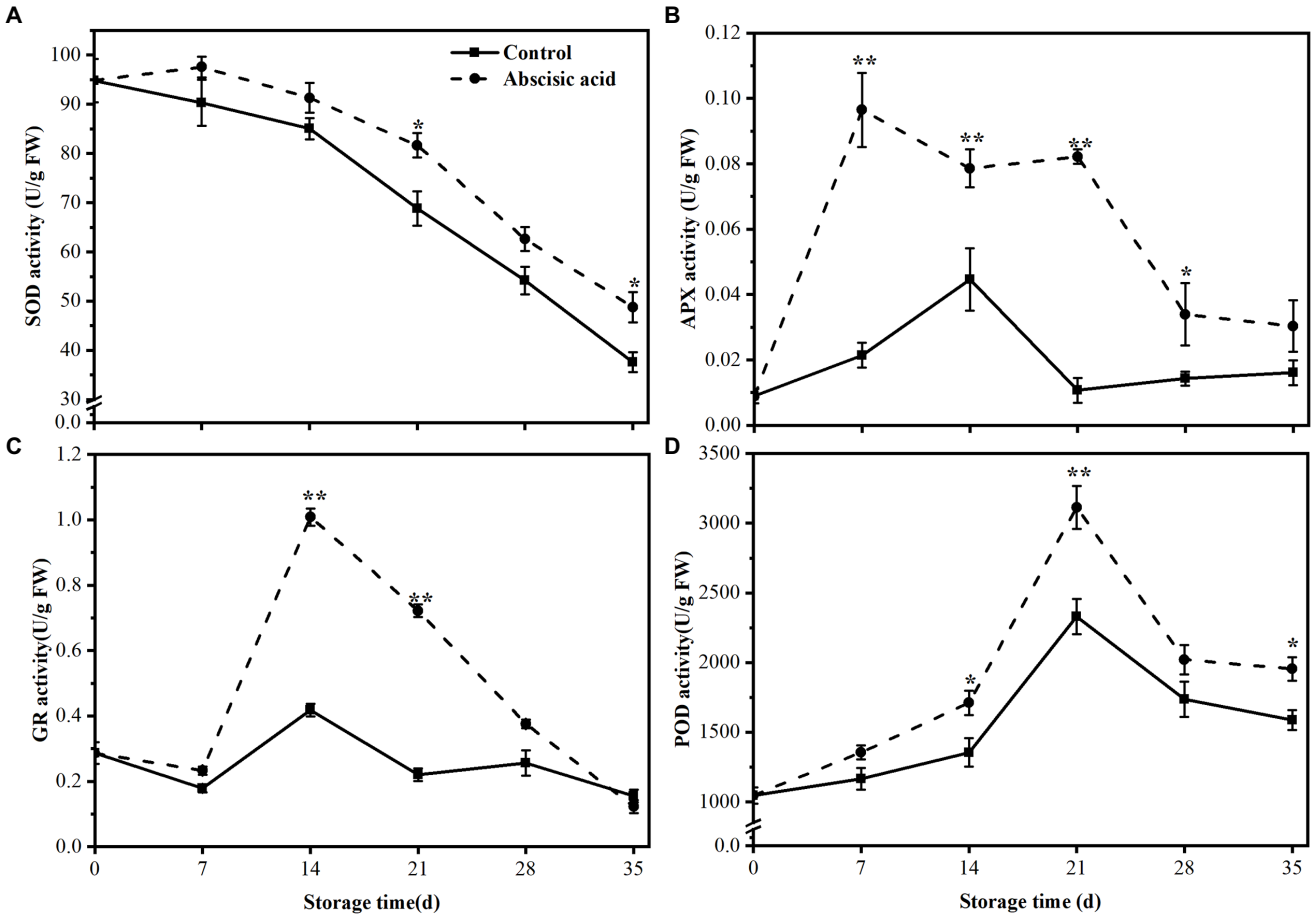
Activity of **(A)** Superoxide dismutase (SOD), **(B)** ascorbate peroxidase (APX), **(C)** glutathione (GR), and **(D)** peroxidase (POD) of peach fruit treated with abscisic acid (ABA) during storage at 4°C. The symbol (*) indicates significant differences among different treatments at *p*   <  0.05. The symbol (**) indicates significant differences among different treatments at *p*  <  0.01.

During cold storage, ROS maybe induced by cold stress and the accumulation of ROS eventually cause plant irreversible injury. One of the most important effects of antioxidant enzymes is to scavenge ROS. Therefore, we detected antioxidant enzyme activities including SOD, POD, APX, and GR. As illustrated in Figure 6A, during the cold storage, the SOD activity showed a decreasing trend during the whole period, regardless of treatment. Moreover, ABA treatment alleviated the decline in SOD activity, especially in the last 3 weeks. The SOD activity in ABA treatment was 16% and 23% higher than that of control at day 21 and day 35, respectively (*p* < 0.05).

Unlike the change of SOD activity, Figure 6B showed that APX enzyme activity of control gradually increased and rapidly decreased at day 14, then maintained a stable trend until the end of storage. On the contrary, the APX activity of ABA-treated fruit was higher than that of control, especially in the first 28 days (*p* < 0.05). In addition, we found that APX activity treated by ABA has risen nearly eight times at day 28. Compared with the control, the GR activity in ABA treatment rose sharply first and reached a peak at day 14. At this time, the activity of GR was 2.43 times higher than that of the control (*p* < 0.05). In the later storage period, GR activity decreased sharply and we found treatment with ABA almost had no effect on GR activity in the last 2 weeks. The variation of GR activity in control fruit was quite small and always remained at a low level (Figure 6C). Finally, the POD activity showed a similar trend regardless of treatment. During the first 21 days of storage, the POD activity increased significantly and subsequently fell until the end of storage. Compared with the control, ABA treatment significantly enhanced the activities of POD (*p* < 0.05), except for day 7 and day 28 (Figure 6D).

## Discussion

For postharvest fruit and vegetables, storage and preservation at low temperature is a wide and common method to extend the shelf life. Low temperature usually causes the occurrence of chilling injury, resulting in fruit quality deterioration and decline in commodity value seriously. The main limiting factor for applying low temperatures to preserve peach fruit is chilling injury. Therefore, it is necessary to seek effective methods to alleviate chilling injury. Previous research has shown that ABA alleviated chilling injury effectively by maintaining cell membrane stability (Guo et al., 2012; Carvajal et al., 2017; Li et al., 2021; Xiong et al., 2021). Our previous study showed that alleviation of chilling injury symptoms in peach fruit by exogenous ABA was associated with the regulation of sucrose metabolism (Zhao et al., 2022). Moreover, Kim et al. (2016) illustrated that in another example of exogenous ABA application leading to improved chilling tolerance, with oriental melon plants, there was an associated increase in endogenous gibberellin (GA_4_) and salicylic acid (SA). Internal browning is a representative symptom of chilling injury and is associated with damage of cell membranes when peach fruit is exposed to improper low temperature conditions (Duan et al., 2022). The results showed that postharvest application of ABA significantly reduced the IB index of peach fruit during storage at 4°C (Figure 1). Based on the findings both in past and present studies together demonstrate that ABA is a promising approach to enhance cold tolerance in diverse fruits and vegetables after harvest.

Fruit softening, ripening, and rotting is mainly controlled by ethylene. As a plant hormone, ethylene is widely involved in physiological and biochemical reactions in plant cells. For example, endogenous ethylene has been implicated in enhancing the cold tolerance of postharvest fruit in earlier investigations (Wei et al., 2019; Yu et al., 2019). In peach, as a typical climacteric fruit, endogenous ethylene and respiration rates increase dramatically during ripening (Hayama et al., 2006; Wang et al., 2017). Ethylene is also broadly involved in plant response to various stressful conditions (Wei et al., 2019). Candan et al. (2006) found that ethylene can increase chilling injury symptoms of plums. Moreover, Pesis et al. (2002) showed that avocado treated with endogenous ethylene caused chilling injury symptoms such as severe pulp browning. 1-aminocyclopropane-1-carboxylic acid (ACC) can be oxidized by ACC oxidase (ACO) to generate ethylene (Hu et al., 2019). As a crucial rate-limiting enzyme in ethylene synthesis, inhibiting the activity of ACO enzyme can effectively reduce ethylene production. In addition, respiration continues after harvesting and increases with tissue deterioration (Li et al., 2022). Respiratory metabolism provided energy for various metabolic processes in plant cells, but also produced large amounts of ROS (del Río et al., 2002). In this research, we found that exogenous ABA effectively reduced ethylene production and respiration rate by inhibiting the expression of genes related to ethylene synthesis, including *PpACO1* and *PpEIN2* (Figures 2, 4). These findings suggest that ethylene is taken part in regulating chilling injury in peach fruit, and that lower ethylene production may help alleviate chilling injury in ABA-treated fruit. At the same time, discrepancies in ethylene responsiveness are likely due to variances in ethylene production as well as ethylene perception machinery abundance.

The imbalance of ROS production usually leads to severe and irreversible damage in plant cells. The occurrence and development of chilling injury are closely related to the excessive accumulation of ROS (Zhao et al., 2021b; Wang et al., 2022). Antioxidant enzymes are the main approach to scavenge excessive ROS and alleviate cell damage induced by oxidative stress, which included SOD, POD, and CAT. Compared with control fruit, the activity of SOD and POD markedly increased in ABA-treated fruit (Figures 6A,D), and this may account for the lower H_2_O_2_ content in ABA-treated fruit (Figure 3). AsA-GSH cycle also takes part in this chemical progress (Tian et al., 2013). Some non-enzymatic substances with antioxidant activity respond to different stress conditions by regulating H_2_O_2_ metabolism (López-Vidal et al., 2016; Piechowiak, 2021). Gao et al. (2016) found that melatonin can effectively reduce the chilling injury index of peaches during cold storage, and this effect may be due to the enhanced activity of antioxidant enzymes thus reducing the accumulation of ROS. Tareen et al. (2012) illustrated that peach fruit treated with SA displayed the highest activity of enzymatic antioxidants, and, in turn, maintained higher quality and showed delayed rotting during storage. Moreover, Cao et al. (2009) showed that loquat fruit treated with methyl jasmonate exhibited lower chilling injury along with higher activities of antioxidant enzymes.

AsA-GSH cycle is also vital for improving the antioxidant capacity of plant cells. Kaya et al. (2021) demonstrated that the AsA-GSH cycle enhanced resistance to environmental stress and delayed senescence of postharvest through scavenging excessive ROS. Figure 6B showed that ABA treatment significantly increased the activity of APX. As an important enzyme for scavenging ROS in the AsA-GSH cycle, APX could catalyze H_2_O_2_ into H_2_O. This finding suggested that the lower H_2_O_2_ content was closely associated with higher APX activity. The presence of both APX and POD allows plants to defend against the toxicity of H_2_O_2_ (Jannatizadeh, 2019). Abscisic acid treatment also increased the activity of GR The effect of ABA on APX and GR activity suggested that ABA is involved in the regulation of the ASA-GSH cycle. Yao et al. (2021) found exogenous GSH treatment could trigger the AsA-GSH cycle and improve antioxidant capacity, therefore alleviating chilling injury in pepper fruits during cold storage. In our study, peach fruit treated with ABA exhibited higher antioxidant enzyme activities, comprising SOD, POD, APX, and GR (Figure 6). This result was consistent with lower H_2_O_2_ content in ABA-treated fruit (Figure 3). All the results suggest that antioxidant capacity is a crucial character for maintaining a balance of ROS. In the present study, we detected the impact of ABA on the transcript abundance level of *PpSOD*, *PpPOD*, *PpCAT*, *PpAPX*, and *PpGR* by using RNA-Seq, and found that the transcript abundance of *PpSOD*, *PpPOD* gene were up-regulated after ABA treatment (Figure 5). This indicates that ABA regulates antioxidant enzyme activity probably by regulating the genes of transcript abundance related to the antioxidant enzyme. Our finding confirms that ABA could eliminate oxidative damage and enhances the antioxidant capacity of plant cells.

## Conclusion

In conclusion, ABA treatment is beneficial in preventing chilling injury and maintaining the commodity quality of peach fruit. The results revealed that treatment with ABA may improve the chilling tolerance of peach fruit by inhibiting the production of ethylene and promoting the scavenging ability of H_2_O_2_. This research clarifies the mechanisms of ABA alleviated chilling injury and provides a theoretical method for applying ABA treatment to maintain a better quality of peach fruit after harvest.

## Data availability statement

The data presented in the study are deposited in the SRA repository, accession number PRJNA866347.

## Author contributions

JT: performed the experiments and analyzed the data, writing and editing. YZ: funding acquisition, conceived the study, and writing–review and editing. SQ: performed the experiments and analyzed the data. QD: methodology, data curation, and investigation. QL: project administration. YD: funding acquisition and validation. All authors contributed to the article and approved the submitted version.

## Funding

This project was supported by the National Natural Science Foundation of China (32102051) and the Agricultural Science and Technology Innovation Program (CAAS-ASTIP2020-IFST-03) from the Chinese Central Government.

## Conflict of interest

The authors declare that the research was conducted in the absence of any commercial or financial relationships that could be construed as a potential conflict of interest.

## Publisher’s note

All claims expressed in this article are solely those of the authors and do not necessarily represent those of their affiliated organizations, or those of the publisher, the editors and the reviewers. Any product that may be evaluated in this article, or claim that may be made by its manufacturer, is not guaranteed or endorsed by the publisher.

## Footnotes

^1^ https://phytozome-next.jgi.doe.gov/info/Ppersica_v2_1
