# Supplementary material for: Abscisic acid alleviates chilling injury in cold-stored peach fruit by regulating ethylene and hydrogen peroxide metabolism
Source: Front Plant Sci. 2022 Sep 6;13:987573. doi: 10.3389/fpls.2022.987573 (PMC9488807; doi:10.3389/fpls.2022.987573)
Supplement: Supplementary file 1 [file Data_Sheet_1.PDF]

Table S1. Primers for RT-qPCR used for analyzing the expression of genes related to ethylene synthesis and signal pathway

Gene	Gene ID	Forward primer sequence	Reverse primer sequence
PpSAMS1	LOC18791900	ACTGCTGCCTATGGGCATTT	AGAGAGCAACAGCAGCACAT
PpSAMS2	LOC18771041	AATCAAACTGCCCTTCACCCC	GAAGGTGTCCATCTCTAAACTGAG
PpACS1	LOC18787058	CCCGAGTTCAGAAAGGCTGT	GGCCAGACAGAACATGACCA
PpACS2	LOC18777197	AGCTGTCGTTGGACTTGGTT	TCCTGCAACAGCCACTTTCA
PpACO1	LOC18784056	CTGGGGCTTCTTTGAGCTTG	GAAGGTGCTTTCCCAGTCCA
PpACO2	LOC18781368	CCCAGTTGTTGACTTGAGCC	TCCCATGGTTCACCAACTCA
PpETR1	LOC18791754	TCCTGGAAAGGGATGCACTG	TCCCTTAGTCACTGACCCGT
PpETR2	LOC18793658	ACGCTTGGCGATACAGGAAA	ACAATTGCACCGCGGAAATC
PpERS1	LOC18768052	TGCGTACTGTCTCGAAACTCA	CTCATCAGGGGGCCATTGTG
PpEIN2	LOC18773117	TCTTCGATTGTGCAGCAGTA	CGCTGAGGTCATAAGGGCAT
PpEIN3	LOC18786295	CAGTCGCCACTCTTATCTTTTTCTC	AGGATACTCAACCCAAACTTCTGA
PpEIN4	LOC18772376	AGGGAAGTTCTCGTCCTGGT	TTGCCTTGCATCATCTGGACA
PpCTR1	LOC18770480	GAGGCCACTTGTACCGATGAT	GGCAGCCATTCACCCAAAAC
PpEIL3	LOC18773761	GTTGGACCTGATATTAGTTCGGATA	TCCTTCCACATTCGTTTCTCCA

Table S2. Primers for RT-qPCR used for analyzing the expression of genes related to antioxidant enzyme pathway and AsA-GSH pathway

Gene	Gene ID	Forward primer sequence	Reverse primer sequence
PpSOD	LOC18789179	ACAACACCACCAAGACATGC	GCCATCTTCTCTGCTGCGTA
PpCAT1	LOC18776773	TCTCATACTGGTCTCAGGCAGA	AAGACAATACAACCGGCGCT
PpAPX2	LOC109950245	CATGCTTCGTTTAGCGTGGC	TTTGGGGTGTTTGGCCTTCA
PpPOD1	LOC18768241	AAAGGGCTTTCGTGGTCCTT	CCGACCCATCACATCCCTGA
PpPOD2	LOC18769960	ACCACTATTGTCCGCAGTGT	TGAAGCATCACATCCGTTGA
PpPOD3	LOC18770065	ACAATGCTACCTGCCCACAA	GGAGGCATCACATCCTCTGAC
PpPOD4	LOC18773443	CATCGGCGCAGCTAAAAACA	CGAAGCATCACAACCCTGGA
PpGR1	LOC18766619	CCCTCCATCTCAGGGGAAGA	GCTGTGTTTTCCAGCACCTG
PpGPX1	LOC18788389	GGGCTCTTTGGAAATGGCATC	GCATACACTTTCTGTTGTCCTTG
PpGPX2	LOC109946232	CCTTAGCCTTGAGCGTGACA	ACAACACACAAAAGCAAGGCA
PpGPX3	LOC18776398	ATTGTGTTGCGTTGCTTTGC	TTCCCTTAGCGTCCTTAACAG
PpGPX4	LOC18766597	AATACCCGTCAACGGCAGAG	ACCTACCAATTATCAACAGTCCC

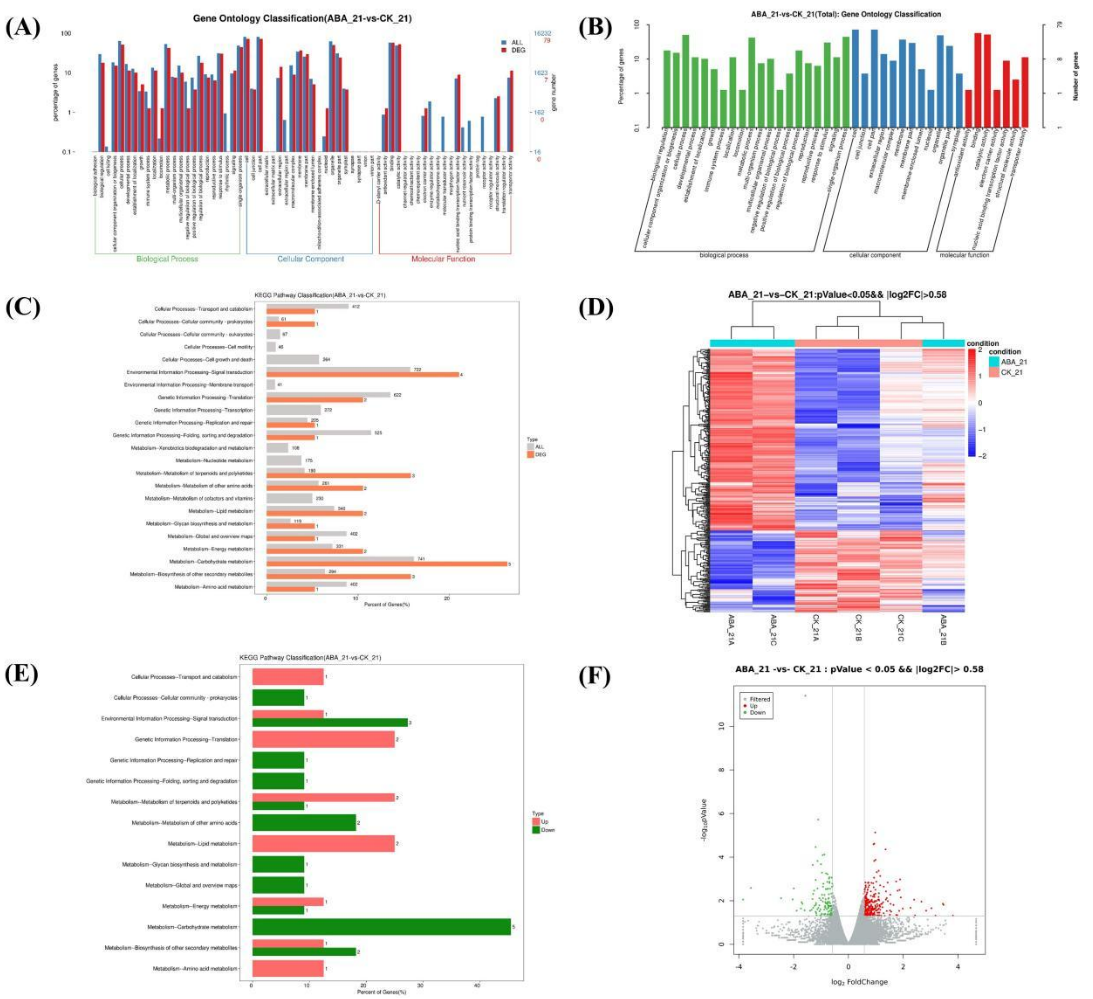

Fig.1: The statistics of RNA-seq data. (A) Gene Ontology (GO) function enrichment of DEGs and all genes in CK21vsABA21; (B) The total representation of Gene Ontology (GO) function enrichment in CK21vsABA21; (C) The gene KEGG function enrichment of all genes and DEGs in CK21vsABA21; (D)ABA_21-vs-CK_21-heatmap-pval-0.05-FC-1.5.gene. (E) Up_vs_Down.KEGG_Classification; (F) ABA_21-vs-CK_21-volcano-pval-0.05-FC-1.5.gene.
